# Hippocampal 1H-MR spectroscopy metabolites are linked to CSF tau pathology in cognitively unimpaired older adults along the Alzheimer’s continuum

**DOI:** 10.1016/j.neurobiolaging.2025.10.005

**Published:** 2025-10-30

**Authors:** Jessica N. Lingad, Casey R. Vanderlip, Sierra Wright, Lorena Sordo, Elizabeth Head, Craig E.L. Stark

**Affiliations:** a University of California, Irvine, School of Biological Sciences, Department of Neurobiology and Behavior, 1400 Biological Sciences III, Irvine, CA 92697, United States; b University of California, Irvine, School of Medicine, Department of Pathology & Laboratory Medicine, Gillespie Neuroscience Research Facility, 837 Health Sciences Rd, Irvine, CA 92697-4540, United States; c University of California, Irvine, School of Medicine, Department of Neurology, Gillespie Neuroscience Research Facility, 837 Health Sciences Rd, Irvine, CA 92697-4540, United States

**Keywords:** Magnetic resonance spectroscopy, Alzheimer’s disease, Aging, Neuroinflammation, Neurometabolism, Biomarkers, Cerebrospinal fluid, magnetic resonance imaging, hippocampus

## Abstract

Relationships between Alzheimer’s disease (AD) pathologies in cognitively unimpaired adults and in vivo neurometabolic properties measured directly from the hippocampus, a vulnerable region early along the AD continuum, are not well-understood in the earliest stages of AD. In a 3T ^1^H-MRS study, we assessed age and AD-related changes in estimates of absolute concentrations of neurometabolites in the right hippocampus. Participants included older adults (age range: 60–85, n = 19) primarily cognitively unimpaired (CU, n = 16), as well as some with cognitive impairment (n = 3). All participants previously received a lumbar puncture for AD disease staging from cerebrospinal fluid (CSF) AD biomarkers (Aβ42, p-tau181 and t-tau), where all were amyloid positive (A+) and most had subthreshold tau pathology (T−). Hippocampal ^1^H-MRS metabolites included total N-acetylaspartate (tNAA), myo-inositol (mIns), total creatine (tCr) and total choline (tCho). Regression analyses were performed for assessing relationships among CSF biomarkers, age, and ^1^H-MRS metabolites measured as tissue-corrected estimates of absolute concentrations (millimoles/kilogram) and as ratios (/tCr and tNAA/mIns). We identified age-related decreases to mIns/tCr, where estimated absolute concentrations revealed that tCr increased while mIns remained stable. Concentrations for tNAA and mIns were positively associated with CSF p-tau181 and t-tau. Levels of tCr and tCho were not associated with any CSF biomarkers. Overall, our results demonstrate that sub-threshold tau pathologies in cognitively unimpaired A+ individuals are associated with hippocampal metabolite changes related to neural metabolism and glial reactivity early in disease progression.

## 1. Introduction

Pathological hallmarks of Alzheimer’s disease (AD) such as the extracellular beta-amyloid (Aβ) plaques and the intraneuronal neurofibrillary tangles comprised of aggregated hyperphosphorylated tau accumulate in the brain decades before individuals exhibit cognitive decline (Jack et al., 2010). Improving our ability to detect how these pathologies alter brain function early along the AD continuum can help identify novel therapeutic targets and broaden patient recruitment for AD clinical trials (Hyman et al., 2012). Proton magnetic resonance spectroscopy (^1^H-MRS) is a non-invasive, *in vivo* technique that has proven useful in measuring neurometabolites known to become altered in aging and AD. Common, readily-assessed metabolites include the neuronal marker N-acetylaspartate (NAA), an amino acid mainly synthesized and stored in neurons (Baslow, 2000) that declines with age, cognitive performance, and in neurodegenerative disorders including AD (Cleeland et al., 2019). Myo-inositol (mIns), an osmolyte primarily found in glia, is a putative marker of glial reactivity and neuroinflammation (Brand et al., 1993; Chang et al., 2013). Total choline (tCho) which includes free choline, glycerophosphocholine, and phosphocholine, is associated with cell membrane turnover as well as inflammation (Cleeland et al., 2019). Increases to mIns and tCho have been associated with reactive gliosis in this disease (Miller et al., 1993; Piersson et al., 2021). Total creatine (tCr) which includes creatine and phosphocreatine is involved in energy metabolism among neurons and glia and is commonly used as an internal reference with the long-standing assumption that tCr is stable over the lifespan and within various neuropathologies. However, recent studies show age- and pathology-related alterations to creatine levels which may confound study findings that normalize to tCr (Li et al., 2003; Gong et al., 2022). Nonetheless, numerous past studies of AD that evaluated metabolite ratios to tCr from within the posterior cingulate cortex (PCC) demonstrate that mIns/tCr and NAA/tCr are associated with disease stage and cognitive impairment. For example, PCC MI/tCr is increased in individuals with Mild Cognitive Impairment (MCI) compared to age-matched controls (Wang et al., 2009) and mIns/tCr is elevated in APOE ε4 carriers before detectable amyloid pathology by CSF Aβ42 (Voevodskaya et al., 2016). Furthermore, several studies report that NAA/tCr is predictive of progression from MCI to dementia (Jessen et al., 2009; Modrego et al., 2011; Seo et al., 2012).

While these detailed characterizations of biochemical changes in response to AD pathologic burden have largely focused on cortical regions such as the PCC, there are a limited number of studies reporting similar changes taking place in the hippocampus. Hippocampal atrophy measured by structural MRI is a well-established biomarker of neurodegeneration in AD, but is often observed after amyloid and medial temporal lobe tau biomarkers become abnormal (Jack et al., 2024), and is not sensitive to detecting early dysfunction. However, hyperactivity of the hippocampus is associated with memory decline and is considered among the most prominent early features of AD prior to clinical diagnosis (Harrison et al., 2016; Tran et al., 2017). Sustained, systemic neuroinflammation is recognized as a prominent early contributor to AD pathogenesis (Leng and Edison, 2021), and histology studies have shown that hippocampal glial reactivity strongly increases with age (David et al., 1997). However, *in vivo* assessments of neuroinflammation of the hippocampus are limited. Given that the criteria for AD diagnosis and staging has been recently revised to use a biological construct, which includes neuroinflammation (Jack et al., 2024), investigating the potential of hippocampal ^1^H-MRS metabolites as early biomarkers of these AD-related pathological changes is of increasing clinical importance.

In this study, we sought to determine how metabolites in the hippocampus estimated as tissue-corrected concentrations (moles/kg of total NAA (tNAA), mIns, tCr, and tCho) or their ratios (tNAA/tCr, mIns/tCr, tCho/tCr, and tNAA/mIns) may reveal associations with age and AD pathologic burden measured from cerebrospinal fluid (CSF) biomarkers of amyloid and tau pathology: Aβ42, phosphorylated tau 181 (p-tau181), and total tau (t-tau) in a cohort primarily comprised cognitively unimpaired older adults early along the AD continuum. We hypothesized that early increases to AD biomarkers are linked to ^1^H-MRS biomarkers of neuronal integrity, neuroinflammation, and brain energy metabolism.

## 2. Materials and methods

### 2.1. Participants

We recruited older adults participating in prospective, longitudinal studies at the UCI Alzheimer’s Disease Research Center (UCI ADRC), a National Institute on Aging (NIA)-funded research center that investigates ways to improve the identification, diagnosis, and treatment of AD and related dementias. Participants at UCI ADRC studies are characterized through extensive neuropsychological testing and clinical examinations during annual visits, where a team of clinicians assign cognitive statuses based on standardized diagnostic criteria (Morris et al., 2006). Here, ADRC participants ranging from cognitively unimpaired (CU) to AD dementia were recruited for a magnetic resonance imaging (MRI) session. Participants were screened for confounding neurological disease or psychiatric illness, spoke fluent English, were right-handed, had normal or corrected-to-normal vision, and had no contraindications for MRI.

Neuroimaging data from participants who previously consented to and received a lumbar puncture (LP) for the characterization of AD biomarkers were selected for analysis in the present study. CSF immunoassays were performed at the UCI ADRC Neuropathology Core using the Roche Elecsys^®^ system for measuring β-amyloid proteinopathy from Aβ42 (Elecsys^®^ beta-Amyloid (1– 42) CSF II), hyperphosphorylated tau from p-tau181 (Elecsys^®^ Phospho-Tau (181P) CSF), and total Tau (t-tau) (Elecsys^®^ Total-Tau CSF). If a participant received more than one LP over their longitudinal participation, the biomarker estimates from the LP closest to the MRI visit was used. The delay between CSF sample collection and subsequent MRI scanning session varied across participants (mean=3.45 ± 2.37 years). Since the samples were frozen, the final measurements were adjusted according to recommendations by a Roche specialist that were based on storage and handling procedures specifically for frozen samples.

All participants provided written informed consent and were compensated for their participation in MRI, neuropsychological testing and for providing CSF in compliance with the University of California, Irvine (UCI) Institutional Review Board (IRB).

### 2.2. Single-voxel ^1^H MR spectroscopy

All MRI and spectroscopy data were collected using a ^1^H 32-channel transmit/receive head coil on a 3T Siemens MAGNETOM Prisma scanner (Siemens Healthineers, Erlangen, Germany) at the UCI Facility for Imaging and Brain Research (FIBRE). Scanning sessions lasted approximately one hour. For anatomical referencing, T1-weighted whole-brain anatomical images were acquired using a 3D magnetization-prepared rapid gradient echo (MP-RAGE). We used the Alzheimer’s Disease Neuroimaging Initiative 3 (ADNI-3) sequence parameters: resolution = 1.0 mm isotropic; TR/TE = 2300/2.98ms; flip angle = 9°.

**Fig. 1.**
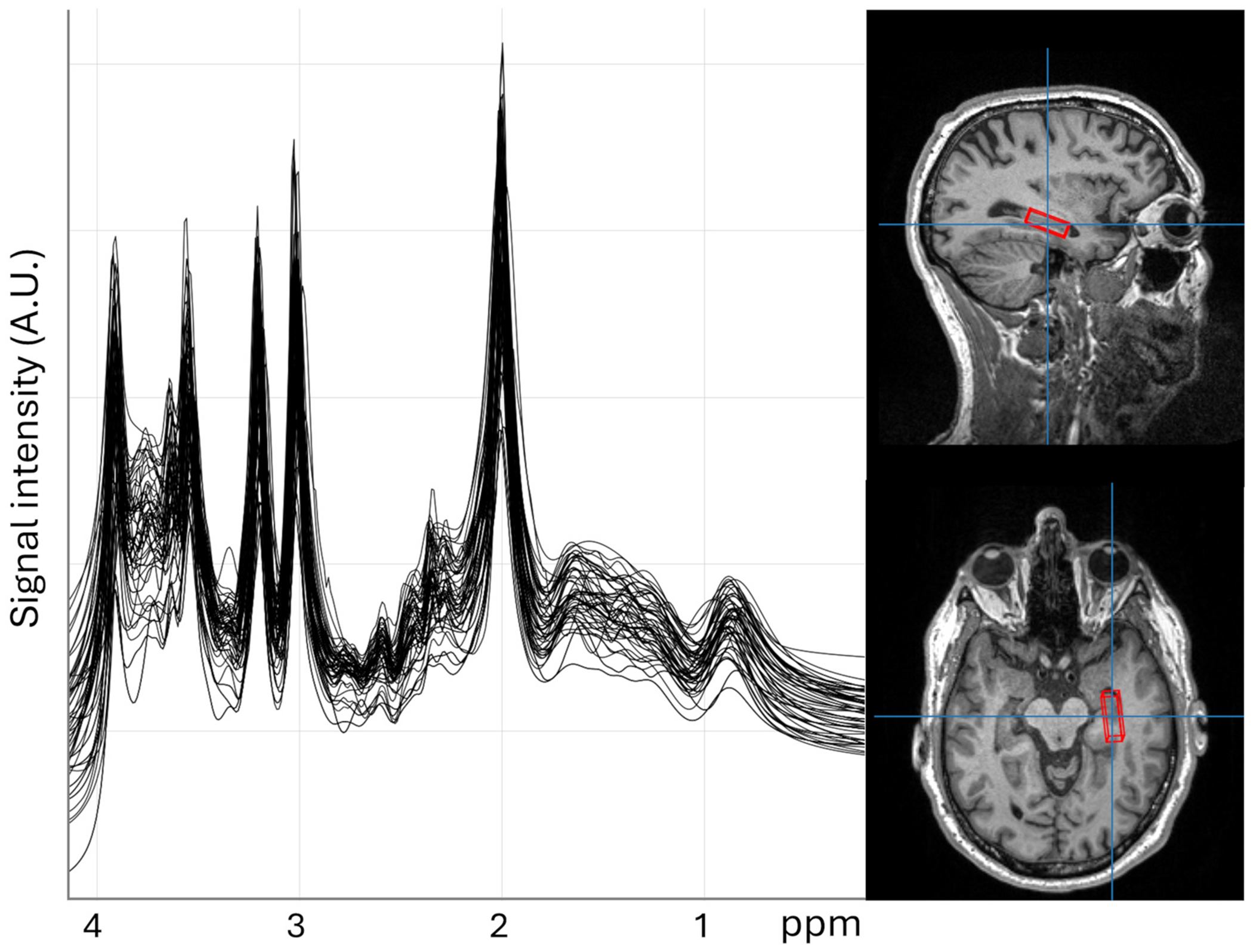
^1^H-MRS was collected from the right hippocampus. All spectra included in the final analysis are shown (n = 19; 1–3 spectra per participant). Sequence: PRESS; 168 averages; 1024 points; TR/TE= 2000/30ms.

The vendor-supplied point-resolved spectroscopy (PRESS) sequence was used for acquiring ^1^H-MRS from a single volume of interest (VOI) aligned to the long axis of the right hippocampus containing mainly the anterior hippocampus referencing to the T1-weighted image or a multislice scout image as an anatomical reference (Fig. 1). We used the following parameters: TR/TE = 2000/30ms; spectral width = 1340 Hz; 1024 complex points; VOI = 2.187 mL (0.9×2.7×0.9 cm^3^); 168 water-suppressed repetitions; 2 water reference repetitions. We used TE= 33ms on one participant to mitigate increased SAR effects when prompted by the scanner system. Water suppression was performed using the Variable Power and Optimized Relaxations delays (VAPOR) method. The vendor-provided automated shimming was applied, as well as manual higher-order shimming to reduce B0 inhomogeneity. We proceeded with ^1^H-MRS acquisition if the system-reported shim resulted in a full-width-half-maximum (FWHM) of 20 Hz or less for the unsuppressed water peak. Following shimming, the PRESS sequence was collected three times consecutively from the same VOI prescription. Four participants were excluded for observed morphologic abnormalities and two participants were excluded for poor VOI alignment to the hippocampus. Full detailed procedures adhering to the minimum reporting standards in MRS studies (Lin et al., 2021) are provided in Supplementary Table 1.

### 2.3. Data processing

The resulting DICOM-structured data files containing each averaged spectrum was exported and was further processed using the open-source Python-based toolbox FSL-MRS (version 2.0.2), part of the FMRIB Software Library (FSL). Expert consensus-recommended methodological practices were implemented (Wilson et al., 2019) using subcommands within the *fsl_mrs_proc* command, such as eddy current correction (*fsl_mrs_proc ecc*), Hankel Lanczos Singular Value Decomposition (HLSVD) for water removal (*fsl_mrs_proc remove*), frequency shift to NAA (*fsl_mrs_proc fshift*) and phase correction of the water-suppressed and unsuppressed spectra (*fsl_mrs_proc phase*) (Clarke et al., 2021). We implemented *svs_segment* which uses FMRIB’s Automated Segmentation Tool (FAST) for tissue segmentation of the T1-weighted image to calculate tissue volume fractions of gray matter (GM), white matter (WM), and CSF within the VOI (Jenkinson et al., 2012). Finally, the *fsl_mrs* command performed fitting of the processed water-suppressed spectrum, referencing to the unsuppressed water reference spectrum and tissue fractions for estimating metabolite concentrations in units of moles per kilogram of tissue water (mol/kg) (Near et al., 2021). The metabolite basis spectra used were simulated and exported from TARQUIN (Wilson et al., 2011). FSL-MRS uses a Linear Combination model where the basis spectra were fitted to the complex-valued spectrum in the frequency domain assuming a Voigt profile to include both Gaussian and Cauchy-Lorentz components. The basis spectra were shifted and broadened with parameters fitted to the data and grouped into 6 metabolite groups: NAA+NAAG, mIns, Cr+PCr, PCh+GPC, and the remaining metabolite basis spectra. A complex polynomial baseline was also concurrently fitted (order=2). Model fitting was performed using the truncated Newton algorithm as implemented in Scipy.

Metabolite concentrations were quantified within the FSL-MRS software and expressed as “absolute units” of mol/kg based on assumptions of tissue-water content and volume fractions. Assumed tissue water densities (g/cm^3^) were 0.78, 0.65, and 0.97 for GM, WM, and CSF, respectively. We used the FSL-MRS default metabolite relaxation times (T2 = 194 ms, T1 = 1.29 s). These fixed relaxation times were applied uniformly across participants; we did not model subject- or age-specific variation in T1 and T2. To validate the effectiveness of partial volume correction, we tested for residual associations between estimated CSF fractions within the VOI and final estimates of absolute metabolite concentrations. None were observed (all p > 0.05, Supplementary Figure 1).

### 2.4. Data quality

Our initial set of metabolites of interest included: tNAA, mIns, tCr, tCho, and the composite measure for glutamate and glutamine (Glx). Metabolite estimates were considered of initial passing quality with Cr SNR ≥ 4. The FSL-MRS algorithm calculated SNR as the ratio of the peak height of the fitted metabolite basis spectrum over the standard deviation of a pure noise region of the spectrum after a matched filter has been applied to both (Clarke et al., 2021). We excluded any spectra with a full width at half maximum (FWHM) of the Cr peak greater than 2.5 standard deviations above the mean Cr FWHM (>15 Hz). While this exceeds previously reported quality thresholds for water linewidth (~13 Hz), ideal linewidths can widely vary across regions (Juchem et al., 2021). Since we collected up to three runs of spectral data per participant, we accounted for variations in spectral quality within participants by performing weighted averages across these runs using the FWHM of each Cr peak, with weights inversely proportional to Cr FWHM. Hence, higher quality spectra with narrower linewidths received greater weight in the final concentrations for each participant’s metabolite estimates, optimizing SNR and using all available data rather than excluding spectra by using overly stringent FWHM cutoffs.

Since the commonly used 20 % threshold for Cramér-Rao lower bound ratio to metabolite concentration (CRLB%) may potentially exclude low- and clinically-relevant metabolite estimates at the individual level (Kreis, 2016), we instead use an alternative approach of testing the reliability of metabolite estimates at the group level to exclude any metabolites with a group mean CRLB% > =20 (Joers et al., 2018; Oz et al., 2021). We identified only Glx having a mean CRLB > = 20 % and excluded this metabolite from further analysis. The final included metabolite estimates for tNAA, mIns, tCr, and tCho had group mean CRLB ratios below 20 %. Individual estimates for metabolite concentrations, CRLB%, and FWHM are shown in Supplementary Figure 2. No relationships were observed between CRLB% and metabolite concentrations.

### 2.5. Statistical analysis

Statistical analyses were performed using the statsmodels module (0.14.1) in Python (3.11.8). We employed ordinary least squares multiple linear regression models to evaluate aging effects on ^1^H-MRS metabolites while covarying for sex (metabolite ~ age + sex), and associations between each CSF biomarker and ^1^H-MRS metabolite including age, sex, and temporal delay in years between LP and MRI visits as covariates (CSF ~ metabolite + age + sex + delay). We also evaluated each model against simpler bivariate models to demonstrate the robustness of any observed associations without covariate adjustments. Models were compared using F-tests (α = 0.05) (Supplementary Table 2). Final statistical inferences and interpretations were drawn from the full model to adequately control for known sources of variance.

For analyses of each CSF biomarker, we applied false discovery rate (FDR) correction using the Benjamini-Hochberg method to control Type I error inflation (α = 0.05) across all four metabolite-age regressions and across the four metabolite predictors (tNAA, mIns, tCr, tCho) for each set of CSF-metabolite regressions. All analyses and corrections were repeated for metabolite ratios tNAA/tCr, mIns/tCr, tCho/tCr, and tNAA/mIns.

## 3. Results

### 3.1. Participant characteristics

Our final sample meeting quality criteria consisted of nineteen participants (12 female, 7 male) with a mean age of 74.7 ± 6.9 years (range: 60–85). The sample was predominantly cognitively unimpaired (CU) (n = 16, 84.2 %) with minimal representation of impaired participants (n = 3: 1 with questionable cognitive impairment (QCI), 1 with mild cognitive impairment (MCI), and 1 with dementia) (Table 1). Based on data-driven ranges of abnormal CSF biomarker thresholds (Dumurgier et al., 2022), our participants were all amyloid-positive (< 963 pg/mL). Five participants (3 CU, 1 MCI, 1 dementia) had positive p-tau181 (> 22 pg/mL). The three CU participants with positive p-tau181 were the only participants also positive for t-tau (>241 pg/mL). These CU participants are thus considered in the early stages of the AD continuum (Jack et al., 2018, 2024). Importantly, since the limited number of impaired participants preclude our ability to conduct meaningful evaluations on cognitive status, we focus our subsequent analyses across the total combined sample and we do not attempt to make statistical inferences about differences across groups, but indicate group membership when plotting data.

### 3.2. Metabolite relationships with age

**Fig. 2.**
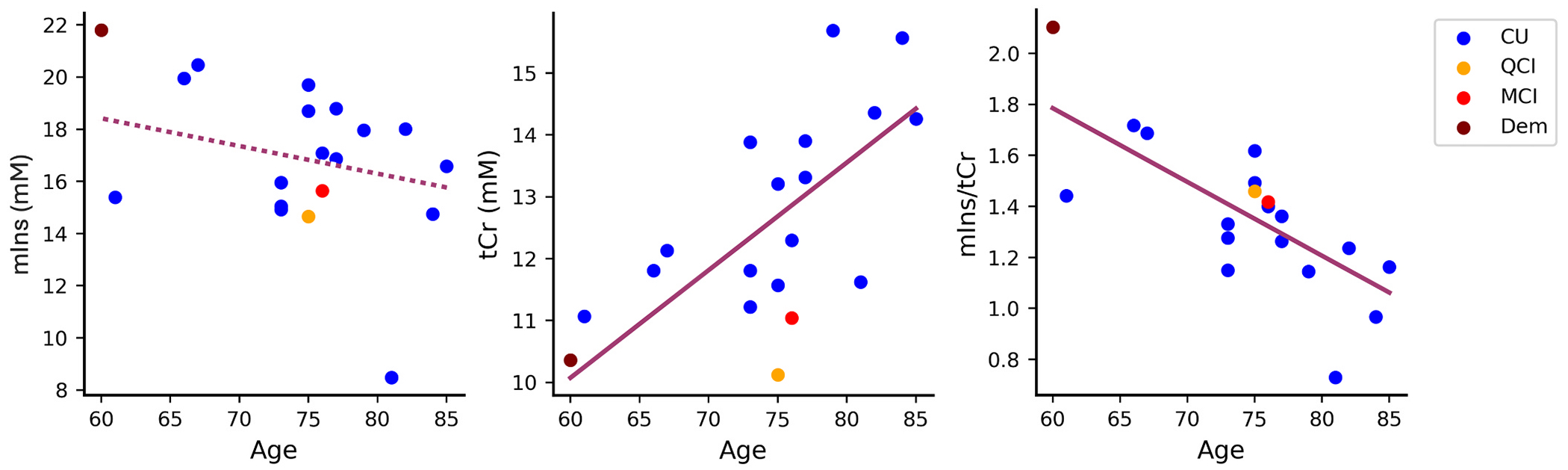
Partial plots showing associations between Age and estimated absolute metabolite concentrations for mIns and tCr, and the mIns/tCr ratio from the model: metabolite ~ age + sex. Purple lines indicate partial correlations for metabolite and CSF biomarker relationships. Significant associations are shown as solid regression lines; the dashed line represents p > 0.05.

We first examined the effect of age on metabolite concentrations while covarying for effects of sex. For the tissue-corrected estimates of absolute concentrations, age only showed a strong positive relationship with tCr (Age β=0.168, 95 % CI [0.076, 0.264], partial r= 0.728, adjusted p = 0.007) (Fig. 2). The mIns/tCr ratio showed the strongest negative association with age (Age β =−0.029, 95 % CI [−0.044, −0.014], partial r = 0.743, adjusted p = 0.004). Age was not reliably associated with estimated absolute concentrations of tNAA, mIns, or tCho in our sample (Supplementary Table 3). Follow-up analyses including CU only (n = 16) were consistent with these findings (Supplementary Figure 3).

### 3.3. CSF AD biomarker relationships with metabolites

**Fig. 3.**
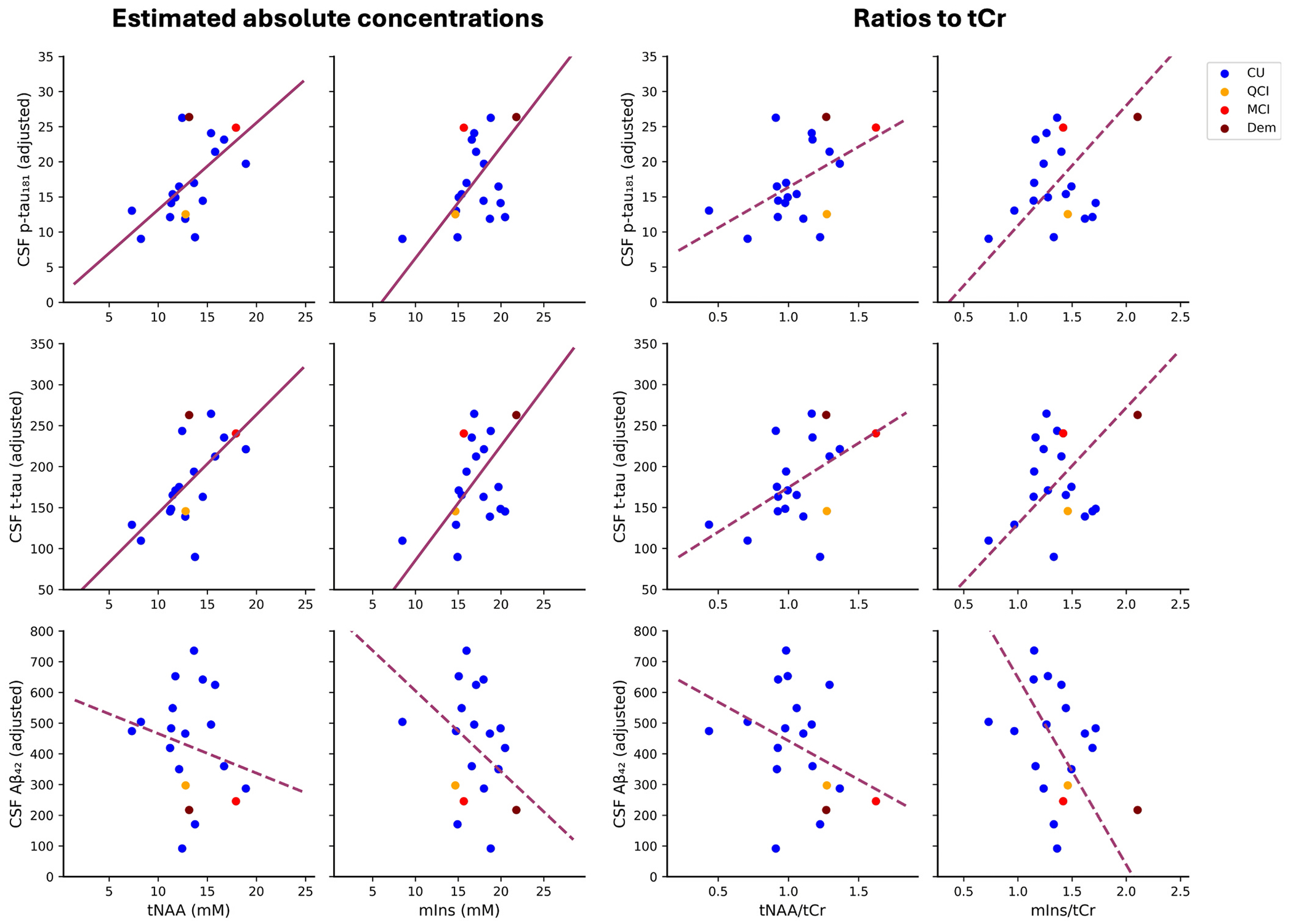
Partial plots showing associations between CSF and hippocampal metabolite concentrations from the model: CSF ~ metabolite + age + sex + LP-MRI delay. The CSF values shown are adjusted for age and sex. Points are colored by diagnostic group: cognitively unimpaired (CU)=blue, questionable cognitive impairment (QCI)=orange, mild cognitive impairment (MCI)=red, dementia (Dem)=maroon. Purple lines indicate partial correlations for metabolite and CSF biomarker relationships. Significant associations are shown as solid regression lines; dashed lines represent relationships that were p *>* 0.05 for both pre- or post-adjustment for multiple comparisons by FDR. Hippocampal tNAA was positively associated with p-tau181 and t-tau. Levels of mIns were also positively associated with p-tau181 and t-tau.

Next, we examined relationships between metabolite concentrations in the hippocampus and three CSF biomarkers of AD, Aβ42, p-tau181, and t-tau (Fig. 3). After controlling for age, sex, and temporal delay, regressions for the tissue-corrected estimates of absolute concentrations showed that average hippocampal tNAA was positively associated with p-tau181 (β=1.237, 95% CI[0.329, 2.146], partial r=0.63, adjusted p = 0.03) and t-tau (β=12.013, 95 % CI[3.943, 20.082], partial r = 0.664, adjusted p = 0.026). Estimated absolute concentrations for mIns were also positively associated with p-tau181 (β=1.584, 95 % CI [0.356, 2.812], partial r = 0.715, adjusted p = 0.030) and t-tau (β=13.993, 95% CI[2.454, 25.531], partial r = 0.693, adjusted p = 0.042). Follow-up analyses for CU only were largely consistent with our positive findings (Supplementary Figure 4). Aβ42 was not associated with any absolute concentration estimates. Ratios to tCr showed similar positive trends for tNAA/tCr with p-tau181 (unadjusted p = 0.048) and t-tau (unadjusted p = 0.042), mIns/tCr with p-tau181 (unadjusted p = 0.045), and the mIns/tCr ratio with Aβ42 (unadjusted p = 0.019). However, all metabolite ratios to tCr as well as tNAA/mIns did not survive multiple comparisons adjustment (Supplementary Table 4).

## 4. Discussion

In the present study, we investigated ^1^H-MRS metabolites in the hippocampus in predominantly CU older adults, hypothesizing that neurochemical properties associated with neuronal integrity, neuroinflammation, and energy metabolism are associated with AD biomarkers early along the AD continuum. Our key findings revealed hippocampal tNAA and mIns, metabolites that are associated with neuronal and glial metabolism, were linked to increased CSF p-tau181 and t-tau but not Aβ42.

It is critical to note that the reliability of absolute concentrations may be affected by age-related decreases to relaxation times recently reported (Hupfeld et al., 2025; Murali-Manohar et al., 2025), especially given our acquisition parameters (TR/TE = 2000/30 ms) which could be considered relatively short (Sporn et al., 2019). Creatine ratios reduce this technical variability through internal referencing, but numerous reports, including our findings in the present study, show creatine increases with age (Cleeland et al., 2019; Gong et al., 2022) which can lead to ambiguity in interpreting aging effects from metabolite ratios. Thus, in this study we used a dual approach of reporting estimates of absolute concentrations as well as creatine ratios to enhance our ability to detect meaningful metabolite alterations.

We observed a positive association between age and estimated absolute concentrations of tCr, a marker of energy metabolism. Since tCr reflects energy metabolism in both neurons and glia, there are numerous possible age-related functional changes related to heightened energy expenditure that may be independent of AD-related pathology (e.g., hyperexcitability, gliosis). Interestingly, we also found that mIns/tCr declined significantly with age, which could initially be interpreted as decreased glial activity. However, the age-related increase in tCr and stable mIns we observed from the absolute estimates indicate that this relationship could be driven by an age-related increase to tCr. These findings exemplify the interpretive complexity of ratio-based measures, but also underscore the importance of future studies to use improved acquisition protocols and quantification methods for accurate absolute quantitation to investigate the underlying biological mechanisms that drive ^1^H-MRS changes.

We found in our moderately-sized sample that tNAA and mIns were associated with increasing CSF p-tau181 and t-tau, suggesting a heightened sensitivity of these ^1^H-MRS biomarkers to specific AD proteinopathies. Ratios to tCr showed evidence of similar trends, providing some supporting evidence for positive associations of tNAA to p-tau181 and t-tau. Interestingly, these novel findings contradict prior studies that report decreases to NAA/Cr in typical aging and in AD and challenge traditional interpretations of NAA as purely reflecting neuronal loss (Cleeland et al., 2019; Piersson et al., 2021). Of the available human studies that examined associations between biochemical properties of the hippocampus and CSF AD biomarkers, findings primarily showed that patients already exhibiting cognitive impairment and advanced AD pathology had reduced NAA associated with CSF AD biomarkers (Jessen et al., 2011; Bittner et al., 2013). As NAA is thought to play a role in enhancing mitochondrial energy production from glutamate (Moffett et al., 2007), we speculate that the novel relationships we observed between increased hippocampal tNAA and CSF p-tau181 and with t-tau may be in part related to increased hippocampal hyperactivity, or more broadly to neuronal dysfunction. A large body of evidence in animal models and human studies have shown that the hippocampus is particularly vulnerable to dysfunctional hyperexcitability in aging and prodromal AD (Bakker et al., 2012; Targa Dias Anastacio et al., 2022; Wilson et al., 2006), and is a phenotype that may even predict tau accumulation (Giorgio et al., 2024). This relationship may reverse in later disease stages as neuronal loss predominates. These findings provide important preliminary evidence for the regional specificity to tNAA alterations and underscores the need for future studies to investigate the basic functional roles of hippocampal tNAA in aging and AD.

Since the hippocampus is one of the early sites for the accumulation of neurofibrillary tangles from hyperphosphorylated tau (Braak and Braak, 1991), our findings of increased mIns potentially capture glial activation that arises before cognitive symptoms emerge. Previous studies have shown that increased neuroinflammation is associated with cognitive decline in AD (Edison et al., 2008; Passamonti et al., 2019) and that heightened glial activation may take place prior to biomarker abnormalities to amyloid and tau (Fan et al., 2017), suggesting mIns may serve as a useful biomarker for the early detection of AD. Notably, we did not observe tNAA/mIns ratios associated with any CSF biomarkers, despite this ratio being a well-established AD marker in other brain regions, likely due to the concomitant increase in both tNAA and mIns.

We found no relationships between tCho and CSF biomarkers, which is surprising given the associations observed between tCho and neuroinflammatory conditions (Chang et al., 2013). Future studies with larger, clinically heterogeneous sample sizes that include alternative brain regions will help to reveal whether mIns and tCho have different temporal dynamics, such as if membrane phospholipid turnover (reflected by tCho) may change on different timescales than energy metabolism or glial activation (reflected by mIns), in different brain regions and/or among different disease states.

Interestingly, CSF Aβ42 was not associated with any estimate of absolute metabolite quantities, but showed modest evidence of a negative association with mIns/tCr. This pattern could be indicative of alterations to the dynamics of neuroinflammatory and bioenergetic processes, but such interpretations remain speculative given the limited statistical evidence. Prior studies examining metabolite ratios from sites with early neocortical Aβ (i.e., PCC) have identified more reliable associations with Aβ42 within CU older adults, but findings are mixed. One study showed only mIns/tCr and not NAA/tCr was associated with CSF Aβ42 positivity in CU participants (Voevodskaya et al., 2016). On the other hand, an investigation of longitudinal changes in CSF Aβ42 did not predict longitudinal mIns/tCr or tNAA/mIns changes in CU older adults (Voevodskaya et al., 2019), and another study examining Aβ accumulation using serial PET in CU participants showed baseline levels of these metabolite ratios associated with increased rate of Aβ accumulation (Nedelska et al., 2017). Differences in spatiotemporal spread between amyloid and tau pathologies could potentially explain this variability in outcomes, where the pattern of Aβ typically involves early neocortical deposition before spreading to allocortex and subcortical regions (Hampel et al., 2021). In contrast, tau aggregation early along the AD continuum takes place in the hippocampus and entorhinal cortex (Braak stage II), sometimes developing prior to amyloid positivity (Therriault et al., 2022). Thus, the hippocampal metabolite alterations we observed may best reflect early pathological changes related to hyperphosphorylated tau and neuronal damage, and less with amyloid.

Our findings also highlight the potential role of ^1^H-MRS metabolites in refining staging models of AD pathology (Jack et al., 2024; Pichet Binette et al., 2025). While CSF and plasma biomarkers provide sensitive indices of amyloid and tau deposition, they lack spatial specificity regarding which neural circuits are most affected at given disease stages (Salvadó et al., 2024). By linking hippocampal metabolites to CSF p-tau181 and t-tau, our results suggest that ^1^H-MRS may add valuable regional specificity to biomarker-based staging frameworks.

Of note, individuals with elevated CSF Aβ42 without tau deposition are at a lower risk for future cognitive decline compared to those with both elevated Aβ42 and tau deposition, suggesting that tau deposition is a key driver of cognitive impairment in AD (Desikan et al., 2012; Moscoso et al., 2025; Ossenkoppele et al., 2022). Our current findings begin to offer a mechanistic link for this relationship between tau deposition and cognitive decline, which suggests that hippocampal neuroinflammation may play a significant role in this process. However, the present study did not examine relationships to memory performance to test this hypothesis, namely due to the cohort being largely cognitively unimpaired and measured at a single time point. Future longitudinal research studies are needed to determine any direct causal relationships between tau deposition, hippocampal neuroinflammation, and cognitive decline.

## 5. Limitations

There was a considerable delay between CSF sample collection and ^1^H-MRS scanning in several participants. If AD pathology has significantly progressed over time, this delay may complicate the interpretations of the relationships between CSF biomarkers and ^1^H-MRS measures. Given the invasive nature of longitudinal CSF sampling, repeat collections were not feasible. We included temporal delay between CSF and MRI sessions as a covariate to account for this potential confound and results remained consistent, but future work in participants with shorter intervals between CSF and ^1^H-MRS data collection will be useful in further uncovering the relationships between AD pathology and hippocampal biochemistry.

The anatomical heterogeneity of the hippocampus and its proximity to the lateral ventricle within the medial temporal lobe pose technical challenges in acquiring reliable ^1^H-MRS data, such as B_0_ magnetic field inhomogeneity and SNR of metabolites of interest (Geurts et al., 2004; Venkatraman et al., 2006). The semiadiabatic LASER sequence (sLASER) has been shown to be robust against the challenges of acquiring reliable spectra from the hippocampus (Wilson et al., 2019; Öz et al., 2021). Furthermore, aging is also associated with decreases to T1 and T2 relaxation times for metabolites and tissue water, which can also differ between regions and bias absolute estimates (Hupfeld et al., 2025; Murali-Manohar et al., 2025). Employing appropriate acquisition parameters (TR, TE) and relaxation constants in our tissue-corrected, absolute quantitation methods will be especially crucial for future studies examining cohort differences, such as in studies comparing different age groups or disease states.

A significant strength of ^1^H-MRS is that it can uniquely and non-invasively allow for examinations of specific biochemical changes in the living brain. Yet, it is important to acknowledge that the metabolites measured by this technique are still indirect indicators of the purported functions mentioned (i.e., neuroinflammation, bioenergetics, and neuronal integrity), since the metabolites measured may participate in more than one physiological process. For example, mIns levels have been linked to glial activation and neuroinflammation, but other mechanisms of mIns exist, such as in osmolyte regulation and calcium signaling (Su et al., 2023). Whether such mechanisms can also influence overall mIns levels independent of neuroinflammation remains to be investigated.

Given emerging evidence that metabolite alterations differ across regions, including hippocampus, posterior cingulate, and other cortical sites (Su et al., 2016; Vints et al., 2022), expanding acquisition beyond a single voxel will be an important next step. Multi-site and multi-region MRS efforts demonstrate the feasibility and value of mapping regional biochemical profiles as a function of biomarker status. Broadening such efforts could reveal whether changes in hippocampal metabolites (e.g. mIns) are part of a global disease state or reflect region-specific vulnerability, providing critical insight into AD pathophysiology.

## 6. Conclusions

Our study highlights age-related changes in hippocampal ^1^H-MRS metabolites and their associations with AD biomarkers. We observed age-related increases to tCr, while elevated CSF p-tau181 and t-tau were associated with higher concentrations of tNAA and mIns. These findings suggest that tau pathology, but not Aβ, plays a significant role in hippocampal neurometabolism prior to cognitive decline. Future work examining longitudinal cognitive changes along with ^1^H-MRS can help identify hippocampal metabolites that are predictive of greater risk for future cognitive impairment in AD.

## Supplementary Material

1

## Figures and Tables

**Table 1:** Participant characteristics. Means, standard deviations, and ranges are shown for all quantitative measures.

Variable	Value
Total Sample	n=19
Sex	
Male	7 (35.0 %)
Female	13 (65.0 %)
Diagnostic Status	
Cognitively Unimpaired	16 (84.2 %)
Questionable Cognitive Impairment	1
Mild Cognitive Impairment	1
Dementia	1
Demographics	
Age (years)	74.47 ± 6.93 [60–85]
Education (years)	17.26 ± 2.23 [12–22]
CSF-MRS Delay (years)	3.47 ± 2.44 [0–7]
CSF Biomarkers	
AB42 (pg/mL)	424.51 ± 186.89 [117.0–747.630]
pTau181 (pg/mL)	17.17 ± 5.93 [8.0–28.43]
Total Tau (pg/mL)	181.95 ± 54.68 [80.0–264.9]
Metabolites	
tNAA (mM)	13.22 ± 2.93 [7.32–18.92]
mIns (mM)	16.88 ± 2.94 [8.48–21.79]
tCr (mM)	12.59 ± 1.66 [10.13–15.69]
tCho (mM)	3.46 ± 0.76 [1.66–4.86]
tNAA/tCr	1.07 ± 0.26 [0.43–1.62]
mIns/tCr	1.37 ± 0.30 [0.73–2.1]
tCho/tCr	0.28 ± 0.06 [0.14–0.40]
tNAA/mIns	0.81 ± 0.19 [0.51–1.15]
VOI Tissue Fraction: CSF	0.11 ± 0.06 [0.03–0.22]
VOI Tissue Fraction: GM	0.67 ± 0.06 [0.56–0.78]
VOI Tissue Fraction: WM	0.22 ± 0.07 [0.12–0.38]

tNAA=total N-acetylaspartate; mIns=myoinositol; tCr=total creatine; tCho=total choline; mM=millimoles/kg; CSF=cerebrospinal fluid; GM=gray matter; WM=white matter.

## Appendix A. Supporting information

Supplementary data associated with this article can be found in the online version at doi:10.1016/j.neurobiolaging.2025.10.005.
