# Supplementary material for: Hippocampal 1H-MR spectroscopy metabolites are linked to CSF tau pathology in cognitively unimpaired older adults along the Alzheimer’s continuum
Source: Neurobiol Aging. Author manuscript; Available in PMC 2026 Jul 17. (PMC13379048; doi:10.1016/j.neurobiolaging.2025.10.005)
Supplement: 1 [file NIHMS2185872-supplement-1.docx]

Supplementary Material

| Site | UCI Facility for Imaging Brain Research (FIBRE) |
| --- | --- |
| 1. Hardware | |
| 1. Field strength | 3T |
| 1. Manufacturer | Siemens |
| 1. Model (software version) | Prisma (VE11C) |
| 1. RF coils: nuclei (transmit/receive), number of channels, type, body part | ^1^H transmit/receive, 32-channel head coil |
| 1. Additional hardware | None |
| 2. Acquisition | |
| 1. Pulse sequence | Point resolved spectroscopy (PRESS) |
| 1. Volume of interest (VOI) locations | Right hippocampus |
| 1. Nominal VOI size | 0.9 x 2.7 x 0.9 cm^3^ |
| 1. Repetition time (TR), echo time (TE) | TR=2000ms, TE=30ms |
| 1. Total number of excitations or acquisitions per spectrum | 168 |
| Additional sequence parameters | |
| 1. Spectral width | 1340 Hz |
| 1. Number of spectral points | 1024 points |
| 1. Water suppression method | Variable Power and Optimized Relaxations delays (VAPOR) |
| 1. Shimming method reference peak, shim acceptance threshold | Vendor-provided automated shimming followed by higher-order shimming on unsuppressed water peak, acceptable water linewidth FWHM *<* 20Hz |
| 3. Data analysis | |
| 1. Analysis software | FSL-MRS 2.0.2 |
| 1. Processing steps deviating from quoted reference or product | None |
| 1. Output measures | Estimated absolute concentrations and ratios to creatine |
| 1. Quantification references and assumptions, model fitting assumptions | Tissue water densities (g/cm^3^):  GM=0.78  WM=0.65  CSF=0.97  Fixed metabolite relaxation times: T2= 194ms  T1= 1.29s  Fitting model basis set (default exported from TARQUIN=27 basis spectra):  Alanine (Ala), aspartate (Asp), creatine (Cr), γ-aminobutyric acid (GABA), glycerophosphocholine (GPC), glucose (Glc), glutamine (Gln), glutathione (Glth), glutamine (Glu), myo-inositol (Ins), lactate (Lac), N-acetyl aspartate (NAA), N-acetyl-aspartyl-glutamate (NAAG), phosphorylcholine (PCh), phosphocreatine (PCr), scyllo-Inositol (Scyllo), taurine (Tau), negative creatine methylene (-CrCH2), lipids (Lip09, Lip13a, Lip13b, Lip20) and macromolecules (MM09, MM12, MM14, MM17, MM20) |
| 4. Data quality | |
| 1. Reported variables | SNR: Ratio of fitted Cr peak height to the SD of a pure noise region  Linewidth: full-width at half-maximum (FWHM Hz) of fitted Cr peak |
| 1. Data exclusion criteria | - Cr SNR < 4 or Cr FWHM >15 Hz - SNR [9.86 ± 2.57], FWHM [10.16 ± 2.83] - 1 participant excluded with Cr FWHM > 15 Hz - Group-level metabolite exclusion: mean CRLB% >20. Excluded: Glx |
| 1. Quality measures of model fitting | Cramér-Rao lower bound relative to estimated metabolite concentration (CRLB%) |
| 1. Sample spectrum | All final included spectra in Figure 1 |

Supplementary Table 1. MRS methods on data acquisition, analysis, and quality in accordance with minimum reporting standards for MRS (Lin et al., 2021).

| 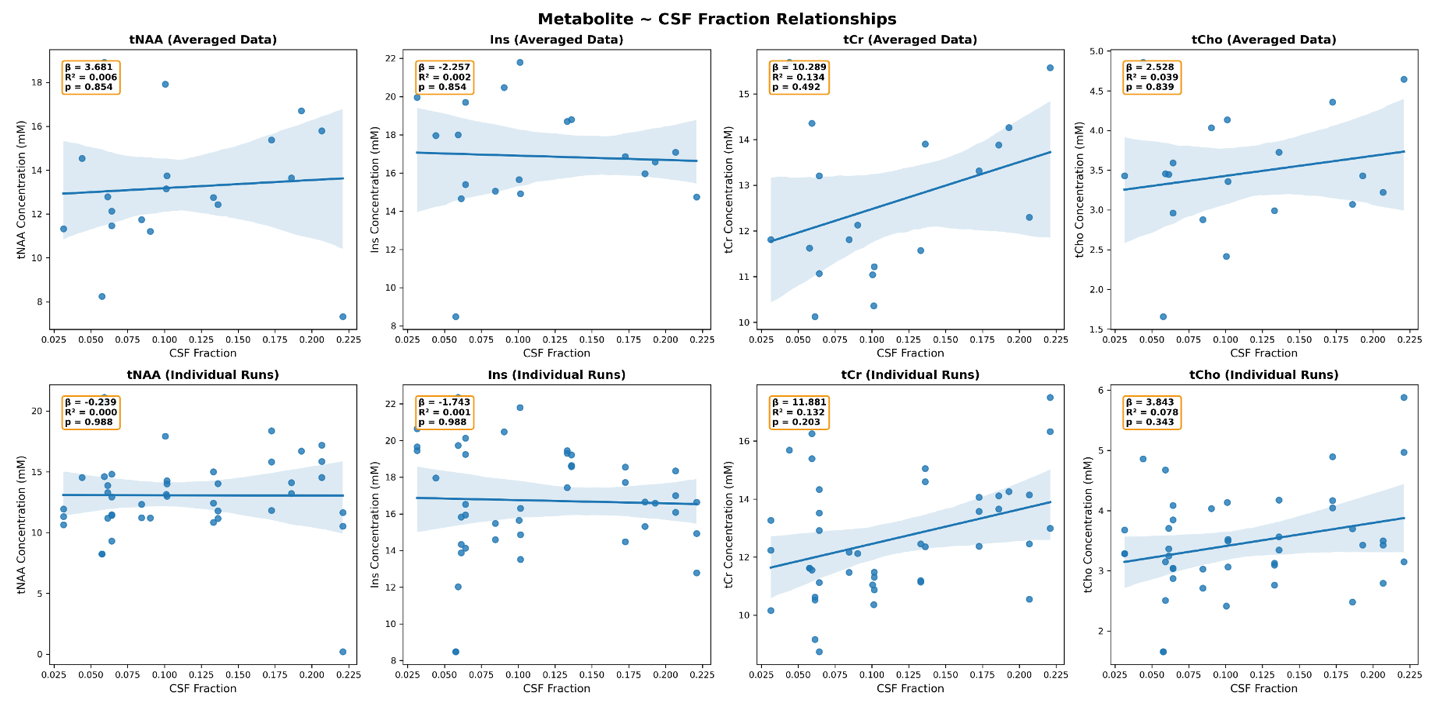  Supplementary Figure 1. Relationships between CSF fraction from within the MRS voxel and final estimated absolute concentrations for final averaged estimates (top row) and from individual runs (bottom row). Clustered standard errors were used to account for repeated measures within participants for analyses of individual runs. |
| --- |

| 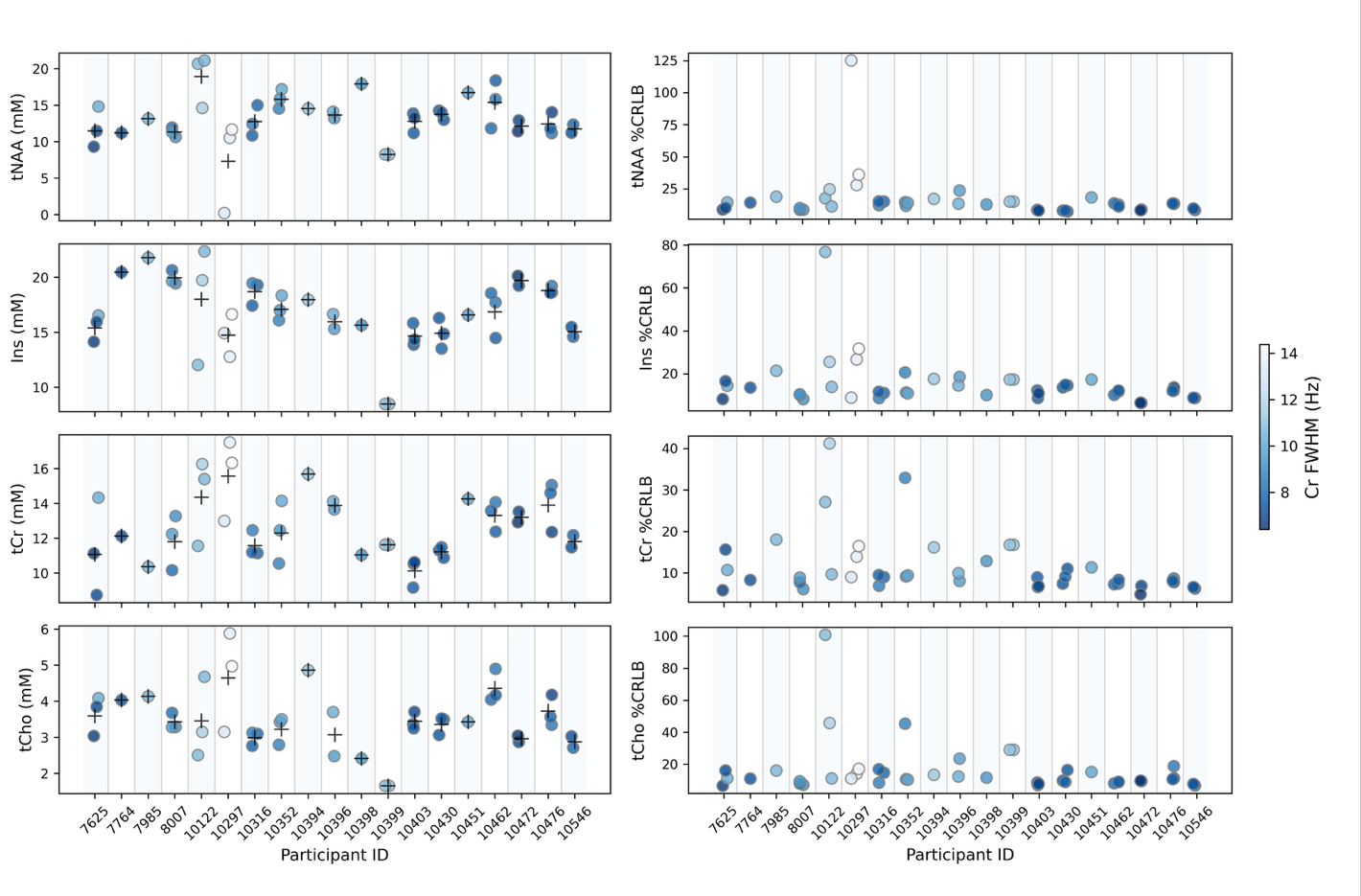  Supplementary Figure 2. Estimated absolute concentrations (left panel) and CRLB% fit quality (right panel) for every participant ranging from 1-3 total spectra collected per participant. Points are indicated by their spectral quality (Cr FWHM), with deeper blues indicating better quality (lower linewidths). Plus symbols (+) in the concentration plots indicate the final weighted averages used in all final regression analyses. |
| --- |

| **Model** | **F-stat** | **F p-value** | **Simple R^2^** | **Full R^2^** | **R^2^ change** | **Simple AIC** | **Full AIC** | **AIC change** |
| --- | --- | --- | --- | --- | --- | --- | --- | --- |
| **mM concentrations** | | | | | | | | |
| ptau181~tNAA | 0.885 | 0.473 | 0.325 | 0.433 | 0.108 | 117.089 | 119.788 | -2.699 |
| ptau181~Ins | 2.145 | 0.140 | 0.139 | 0.410 | 0.271 | 121.725 | 120.539 | 1.185 |
| ptau181~tCr | 0.135 | 0.937 | 0.089 | 0.115 | 0.026 | 122.786 | 128.243 | -5.457 |
| ptau181~tCho | 0.460 | 0.715 | 0.039 | 0.125 | 0.086 | 123.818 | 128.033 | -4.215 |
| ttau~tNAA | 0.746 | 0.543 | 0.389 | 0.473 | 0.084 | 199.595 | 202.779 | -3.184 |
| ttau~mIns | 2.065 | 0.151 | 0.114 | 0.386 | 0.272 | 206.645 | 205.683 | 0.962 |
| ttau~tCr | 0.065 | 0.978 | 0.111 | 0.123 | 0.012 | 206.722 | 212.459 | -5.737 |
| ttau~tCho | 0.473 | 0.706 | 0.035 | 0.124 | 0.089 | 208.281 | 212.446 | -4.164 |
| ab42~tNAA | 0.516 | 0.678 | 0.042 | 0.138 | 0.095 | 254.830 | 258.837 | -4.007 |
| ab42~mIns | 0.959 | 0.439 | 0.023 | 0.190 | 0.167 | 255.211 | 257.659 | -2.447 |
| ab42~tCr | 0.336 | 0.800 | 0.134 | 0.192 | 0.058 | 252.915 | 257.595 | -4.679 |
| ab42~tCho | 0.497 | 0.690 | 0.004 | 0.100 | 0.096 | 255.573 | 259.649 | -4.077 |
| **ratios** | | | | | | | | |
| ptau181~tNAA/tCr | 1.25 | 0.33 | 0.13 | 0.32 | 0.18 | 121.82 | 123.31 | -1.49 |
| ptau181~mIns/tCr | 2.10 | 0.15 | 0.02 | 0.32 | 0.31 | 124.24 | 123.17 | 1.07 |
| ptau181~tNAA/mIns | 0.57 | 0.64 | 0.06 | 0.16 | 0.10 | 123.42 | 127.22 | -3.80 |
| ptau181~tCho/tCr | 0.65 | 0.59 | 0.00 | 0.12 | 0.12 | 124.55 | 128.06 | -3.51 |
| ttau~tNAA/tCr | 1.26 | 0.33 | 0.15 | 0.33 | 0.18 | 205.89 | 207.35 | -1.46 |
| ttau~Ins/tCr | 1.77 | 0.20 | 0.01 | 0.28 | 0.27 | 208.85 | 208.73 | 0.12 |
| ttau~tNAA/mIns | 0.58 | 0.64 | 0.10 | 0.20 | 0.10 | 206.88 | 210.67 | -3.79 |
| ttau~tCho/tCr | 0.62 | 0.61 | 0.0001 | 0.12 | 0.12 | 208.95 | 212.58 | -3.64 |
| ab42 ~tNAA/tCr | 0.25 | 0.86 | 0.17 | 0.21 | 0.04 | 252.12 | 257.14 | -5.02 |
| ab42~mIns/tCr | 2.19 | 0.13 | 0.12 | 0.40 | 0.28 | 253.27 | 251.95 | 1.32 |
| ab42~tNAA/Ins | 0.47 | 0.71 | 0.02 | 0.11 | 0.09 | 255.36 | 259.52 | -4.16 |
| ab42~tCho/tCr | 0.58 | 0.64 | 0.05 | 0.15 | 0.10 | 254.69 | 258.47 | -3.78 |

Supplementary Table 2. F-test results [F(3,14)] comparing the full model including all covariates (“Full” = CSF~Metabolite+Age+Sex+Delay) versus the reduced CSF-metabolite model (“Simple” = CSF~Metabolite). None of the simpler models had any meaningful improvements compared to the full models that included covariates. AIC=Akaike Information Criterion.

| **Metabolite** | **β Age** | **β Age p-value** | **β Age FDR p-value** | **β Sex** | **β Sex p-value** | **β Sex FDR p-value** |
| --- | --- | --- | --- | --- | --- | --- |
| tNAA | 0.049 | 0.677 | 0.903 | -1.03 | 0.541 | 0.541 |
| mIns | -0.10 | 0.270 | 0.539 | 2.58 | 0.074 | 0.296 |
| tCr | 0.17 | 0.002** | 0.007** | 0.52 | 0.422 | 0.541 |
| tCho | 0.003 | 0.912 | 0.912 | 0.45 | 0.300 | 0.541 |
| tNAA/tCr | -0.01 | 0.299 | 0.298 | -0.14 | 0.305 | 0.407 |
| mIns/tCr | -0.02 | 0.001** | 0.004** | 0.14 | 0.162 | 0.324 |
| tCho/tCr | -0.004 | 0.026 | 0.052 | 0.01 | 0.584 | 0.584 |
| tNAA/mIns | 0.007 | 0.221 | 0.294 | 0.18 | 0.047 | 0.188 |

Supplementary Table 3. Beta coefficients for the regression models predicting metabolite concentrations using Age and Sex as predictor variables. *p<0.05, **p<0.01.

| **Comparison** | **β Coeff.** | **CI lower** | **CI upper** | **partial R** | **P original** | **P FDR** |
| --- | --- | --- | --- | --- | --- | --- |
| ptau181~tNAA | 1.237 | 0.329 | 2.146 | 0.630 | 0.011* | 0.030* |
| ptau181~Ins | 1.584 | 0.356 | 2.812 | 0.715 | 0.015* | 0.030* |
| ptau181~tCr | 0.805 | -1.798 | 3.408 | 0.233 | 0.518 | 0.518 |
| ptau181~tCho | 1.621 | -2.866 | 6.107 | 0.217 | 0.451 | 0.518 |
| ttau~tNAA | 12.013 | 3.943 | 20.082 | 0.664 | 0.007** | 0.026* |
| ttau~Ins | 13.993 | 2.454 | 25.531 | 0.693 | 0.021* | 0.042* |
| ttau~tCr | 8.153 | -15.722 | 32.028 | 0.256 | 0.476 | 0.476 |
| ttau~tCho | 14.260 | -27.102 | 55.623 | 0.207 | 0.472 | 0.476 |
| ab42~tNAA | -12.864 | -48.143 | 22.415 | -0.212 | 0.447 | 0.596 |
| ab42~Ins | -26.268 | -71.574 | 19.039 | -0.417 | 0.234 | 0.468 |
| ab42~tCr | 46.166 | -32.140 | 124.471 | 0.416 | 0.227 | 0.468 |
| ab42~tCho | 0.084 | -143.164 | 143.333 | 0.0004 | 0.999 | 0.999 |
| ptau181~tNAA/tCr | 11.536 | 0.144 | 22.927 | 0.521 | 0.048* | 0.095 |
| ptau181~Ins/tCr | 17.116 | 0.454 | 33.777 | 0.723 | 0.045* | 0.095 |
| ptau181~tNAA/Ins | 10.721 | -9.920 | 31.361 | 0.359 | 0.284 | 0.379 |
| ptau181~tCho/tCr | 24.900 | -45.386 | 95.186 | 0.255 | 0.460 | 0.460 |
| ttau~tNAA/tCr | 108.613 | 4.603 | 212.622 | 0.533 | 0.042* | 0.151 |
| ttau~Ins/tCr | 141.745 | -16.566 | 300.057 | 0.674 | 0.075 | 0.151 |
| ttau~tNAA/Ins | 121.471 | -64.095 | 307.037 | 0.436 | 0.182 | 0.243 |
| ttau~tCho/tCr | 201.285 | -448.613 | 851.183 | 0.224 | 0.517 | 0.517 |
| ab42 ~tNAA/tCr | -252.675 | -638.214 | 132.864 | -0.368 | 0.182 | 0.363 |
| ab42 ~Ins/tCr | -608.856 | -1102.517 | -115.194 | -0.782 | 0.019* | 0.077 |
| ab42 ~tNAA/Ins | -96.407 | -767.524 | 574.709 | -0.106 | 0.763 | 0.763 |
| ab42 ~tCho/tCr | -958.520 | -3132.751 | 1215.711 | -0.312 | 0.360 | 0.481 |

Supplementary Table 4. Regression coefficients for metabolite associations with CSF AD biomarkers from the model CSF ~ Metabolite + Sex + Age + Delay. *p<0.05, **p<0.01.

| 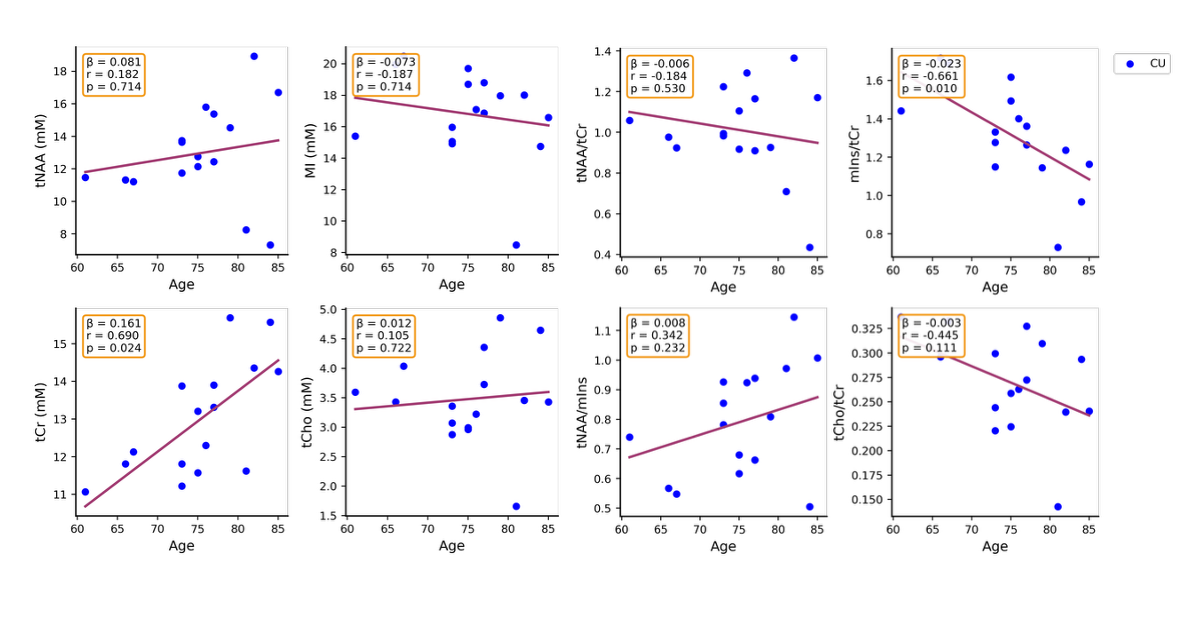  Supplementary Figure 3. Metabolite-age partial plots for cognitively unimpaired (CU) participants only (n=16). Regression coefficients, partial r, and unadjusted p-values are shown. |
| --- |

| 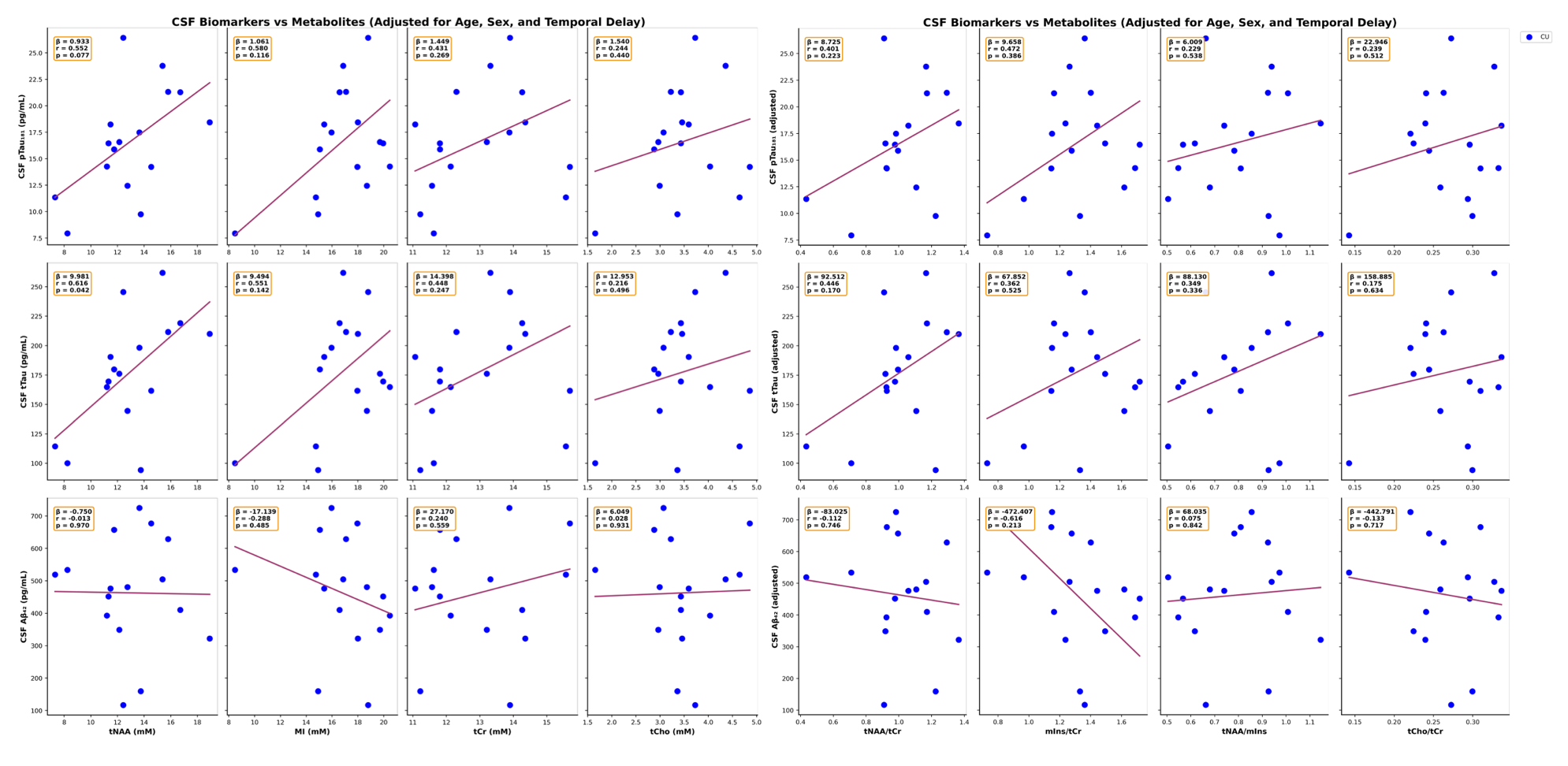  Supplementary Figure 4. CSF-metabolite partial plots for cognitively unimpaired (CU) participants only (n=16). Regression coefficients, partial r, and unadjusted p-values are shown. |
| --- |
